# Case Report: Radicular Amputation as an Alternative to Tooth Extraction

**DOI:** 10.1155/crid/1204886

**Published:** 2026-07-20

**Authors:** Alejandro Álvarez-Sagües, Cristina Pérez, Azogue Ramos

**Affiliations:** ^1^ Dentistry Department, Faculty of Medicine, San Pablo CEU University, Madrid, Spain

**Keywords:** endodontic retreatment, root amputation, root resection, vertical root fracture

## Abstract

Vertical root fracture represents a challenging clinical condition that is frequently associated with poor prognosis and often leads to tooth extraction as the treatment of choice. However, alternative therapeutic approaches aimed at preserving natural dentition should be considered. This report presents a clinical case of root amputation in a mandibular molar 4.6, which was diagnosed as a complete vertical root fracture based on clinical and radiographic findings. As an alternative to extraction, root amputation of the affected mesial root was performed. The evaluation of this treatment modality remains challenging due to the heterogeneity of clinical criteria and indications reported in the literature. Nevertheless, root amputation may represent a favorable therapeutic option in selected cases, particularly in molars presenting with a localized vertical root fracture involving a single root. This case highlights the potential of conservative surgical approaches to achieve long‐term preservation.

## 1. Introduction

In contemporary dental practice, the preservation of dentinal and periradicular tissues represents a primary therapeutic objective. Among the procedures aimed at achieving this goal, endodontic therapy constitutes a fundamental component. Endodontics is concerned with the study, diagnosis, and management of pulpal anatomy and its associated pathological conditions.

Endodontic treatment involves both mechanical and chemical phases. The mechanical phase includes the use of hand files and rotary instrumentation systems to achieve three‐dimensional shaping of the root canal system. The chemical phase is primarily based on irrigation, which facilitates access to and disinfection of the complex root canal anatomy, including ramifications, isthmuses, apical deltas, and lateral canals [1]. Sodium hypochlorite remains the irrigant of choice and is used under varying conditions of concentration, exposure time, and temperature [1].

Endodontic therapy has been shown to yield high success rates, reaching approximately 96% in vital teeth and about 87% in necrotic cases, where the bacterial load is typically higher [2] (Figure 1). Therefore, when conducted following appropriate clinical protocols and with adequate technical execution, endodontic treatment offers a highly predictable prognosis [1, 2].

**Figure 1:**
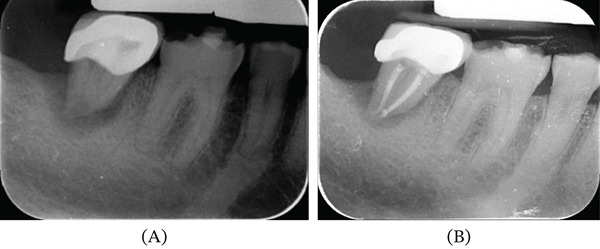
(A) Chronic apical periodontitis of Tooth 4.7. (B) One‐year follow‐up after endodontic treatment of Tooth 4.7, showing evidence of healing.

The success of endodontic therapy is closely related to the subsequent restorative phase, which is essential for restoring both masticatory function and esthetics.

Following completion of root canal treatment, one of the most challenging complications that may arise is the occurrence of vertical root fractures. These fractures can develop over time or because of occlusal overload. Maxillary premolars and the mesial roots of mandibular molars have been reported to be the most frequently affected teeth [3] (Figure 2).

**Figure 2:**
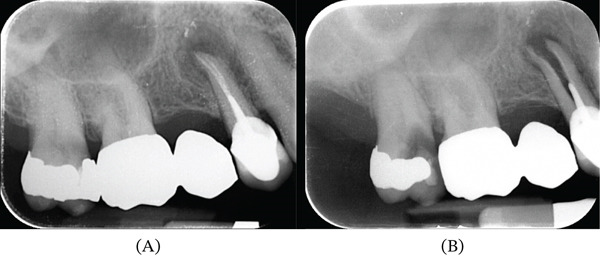
(A) December 2015: apicolateral lesion of tooth 1.4 functioning as a bridge abutment with a metallic post, diagnosed as a complete vertical root fracture. (B) September 2017 follow‐up of Tooth 1.4 showing evidence of complete root fracture.

The etiology of vertical root fractures is considered multifactorial. Predisposing factors include the anatomical configuration of the affected tooth, the presence of carious lesions, a history of dental trauma, and the degree of coronal structure loss associated with extensive restorative procedures. Additional contributing factors include parafunctional habits, such as bruxism, diminished alveolar bone support secondary to periodontal disease, and age‐related structural changes. Iatrogenic factors may also be involved, encompassing both endodontic causes—such as overinstrumentation of the root canal system—and restorative factors, including inappropriate selection of the restorative strategy or improper placement of intraradicular posts, whether due to unjustified indication or inadequate post length [3, 4] (Figure 2).

The etiology of vertical root fractures is typically multifactorial, resulting from the interaction of one or more predisposing and contributing factors. In the majority of cases, the presence of a vertical root crack or fracture leads to tooth extraction as the treatment of choice. However, the potential advantages of tooth preservation through root amputation are frequently underestimated. According to the American Academy of Periodontology, root amputation [5] is defined as the surgical removal of one or more roots of a tooth, with or without excision of the associated coronal portion [5].

This approach enables the preservation of teeth that would otherwise be deemed nonrestorable and indicated for extraction. Following the procedure, appropriate restoration of the remaining tooth structure is generally required, ensuring periodontal stability and compliance with established restorative principles [6]. Root amputation is also indicated in the management of periodontal disease and furcation involvement in molars, particularly in cases presenting with significant bone loss affecting one of the roots [6]. Consequently, this therapeutic option provides patient‐centered benefits, such as preserving the tooth and stimulating natural bone regeneration.

## 2. Objective

The aim of this report is to emphasize root amputation as a predictable and conservative therapeutic alternative to tooth extraction, demonstrating its value in maintaining natural dentition and supporting long‐term oral function when proper case selection and technique are applied.

## 3. Case Report

This case report has been written according to Preferred Reporting Items for Case reports in Endodontics (PRICE) 2020 Guidelines (Figure 3) [7].

**Figure 3:**
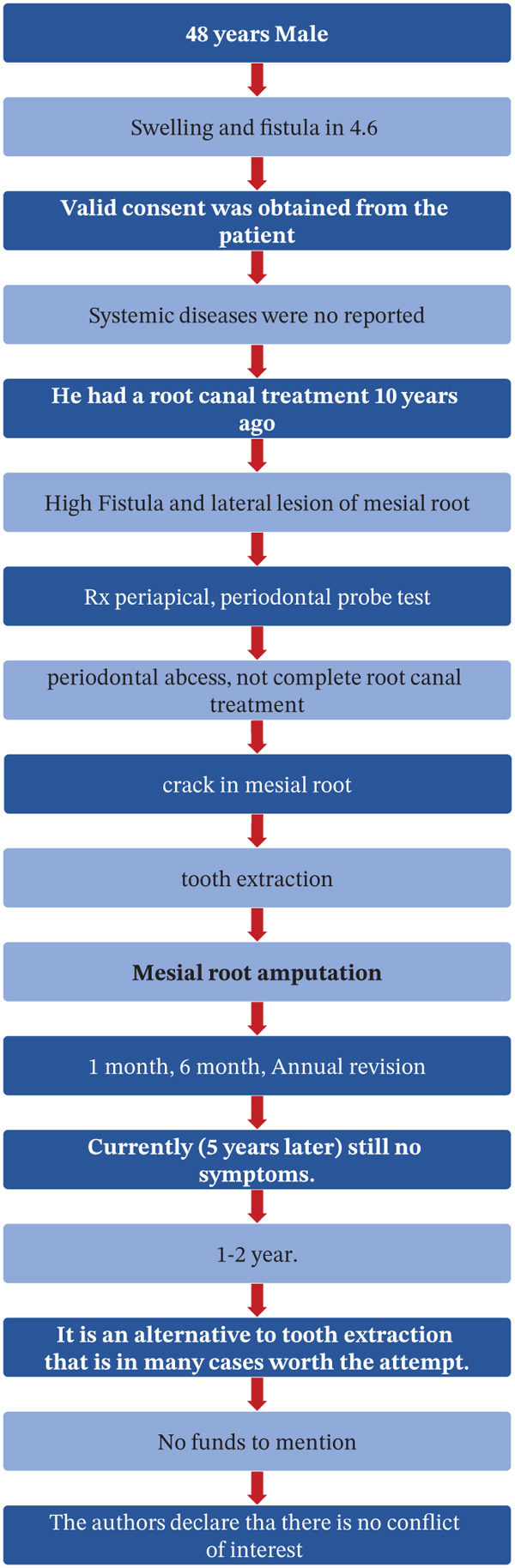
PRICE 2020 flowchart (Nagendrababu et al. 2020) [7] showing the steps involved in the case report.

A 48‐year‐old male patient, with no relevant medical history or known drug allergies, presented with a vestibular sinus tract located at the coronal aspect of Tooth 4.6 (Figure 4), with no associated symptoms of pain. The tooth had a history of endodontic treatment performed more than 10 years earlier.

**Figure 4:**
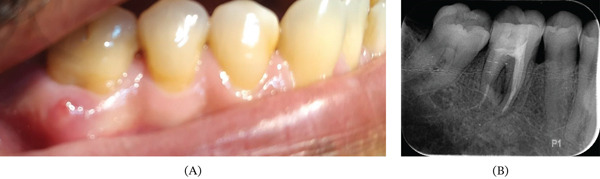
(A) Clinical examination of Tooth 4.6 showing a coronal fistula with localized vestibular inflammation. (B) Diagnostic periapical radiograph of Tooth 4.6, showing a lateral lesion in the middle third of the mesial root.

The diagnosis of vertical root fracture was established based on a comprehensive assessment of clinical and radiographic findings. It should be noted that, unfortunately, cone‐beam computed tomography (CBCT) was not available at the time of initial diagnosis. Fistulography demonstrated a sinus tract extending toward the middle third of the mesial root, which corresponded with the radiolucent area observed on the diagnostic periapical radiograph, further supporting an endodontic origin. Clinically, the tooth exhibited negative responses to percussion and palpation, a positive response to mastication testing, and a localized periodontal probing defect associated with the mesial root. These findings were highly suggestive of a complete vertical root fracture.

A differential diagnosis was considered, including endodontic failure, isolated periodontal defects, and combined endodontic–periodontal lesions. However, the presence of a localized deep probing defect in conjunction with a sinus tract and only pain on mastication strongly supported the diagnosis of vertical root fracture rather than other pathological conditions.

Following a detailed explanation of the treatment plan, written informed consent was obtained before the intervention.

The procedure was performed under local infiltrative anesthesia using 4% articaine with epinephrine (1:100,000); one carpule was administered via vestibular infiltration with additional reinforcement from the lingual. A full‐thickness mucoperiosteal vestibular flap was elevated through a sulcular incision using a No. 15 scalpel blade (Swann–Morton) with appropriate releasing incisions, providing adequate access and visibility.

Upon flap reflection, a fracture line was directly visualized on the mesial root, associated with an isolated vertical bone defect that corresponded to the previously localized periodontal probing defect. The fracture line was further confirmed using methylene blue dye.

Root amputation was performed using a GSD1 diamond root‐sectioning bur under irrigation of saline solution. Due to root fusion, both the mesiobuccal and mesiolingual components of the mesial root were resected (Figure 5). The coronal structure was recontoured using an Arkansas stone bur in a contra‐angle handpiece to eliminate irregularities. A residual coronal portion of approximately 2–3 mm was intentionally preserved to maintain soft tissue support and promote interdental papilla stability. The flap was repositioned and secured with 5‐0 silk sutures.

**Figure 5:**
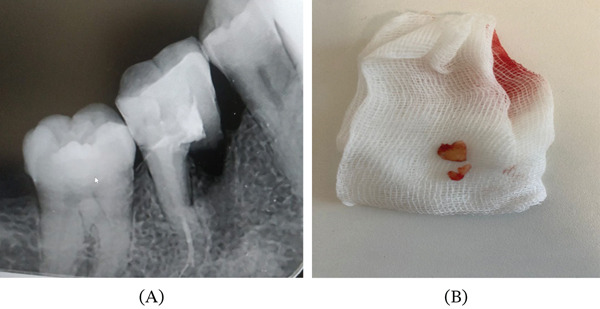
(A) Periapical radiograph of Tooth 4.6 following amputation of both the mesiobuccal and mesiolingual roots. (B) Root amputation performed on the mesial root of Tooth 4.6.

Following the procedure, analgesic therapy with dexketoprofen (Enantyum) was prescribed, starting 2 h after surgery and thereafter on demand (6–8 h), together with antibiotic coverage consisting of amoxicillin 500 mg/8 h for 4 days. At 1 week following suture removal, minor occlusal adjustment was carried out to reduce mesial loading, whereas the existing restoration was left unaltered.

The patient did not attend follow‐up visits at our institution until 4 years after the surgical intervention. During this interval, he reported maintaining regular annual dental examinations and professional prophylaxis.

At the 4‐year follow‐up, the patient was asymptomatic, with no clinical or radiographic evidence of pathology. Periapical radiography and CBCT confirmed the absence of clinical symptoms and the stability of the treatment outcome (Figures 6 and 7).

**Figure 6:**
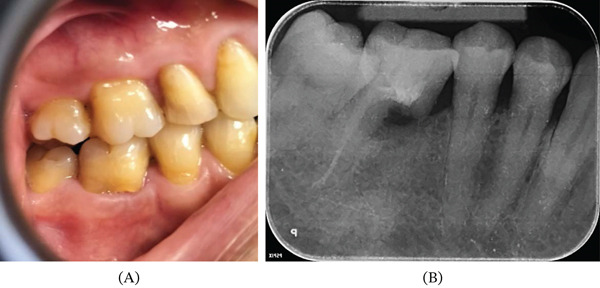
(A) Clinical follow‐up examination 4 years after treatment. (B) Periapical radiograph of Tooth 4.6 after 4 years showing bone regeneration and absence of radiolucent lesions.

**Figure 7:**
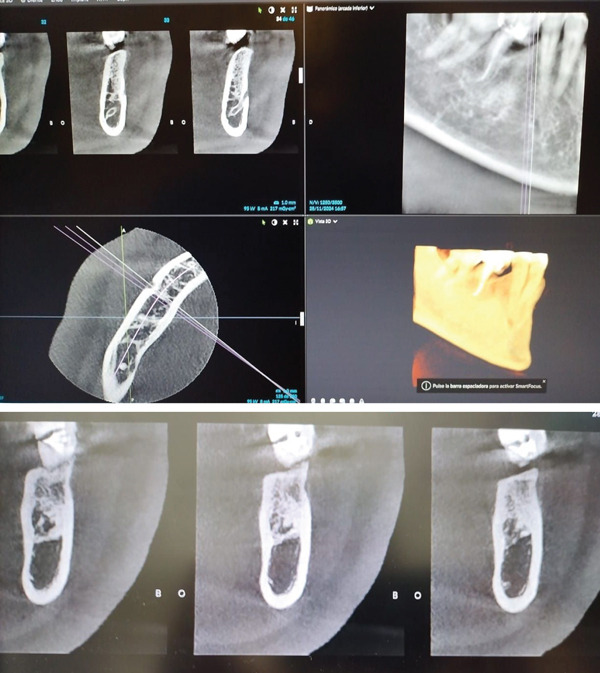
CBCT of the fourth quadrant showing, in the transverse sections at the level of the mesial root amputation, both vertical and horizontal bone regeneration associated with Tooth 4.6.

## 4. Discussion

Root amputation techniques are primarily employed in teeth presenting postendodontic, periodontal, or combined pathologies. The first documented case of root amputation was published in 1884 by Farrar [8], who described the procedure as a “radical and heroic treatment” [8].

Corbella et al. [9] conducted a systematic review in 2022 across multiple databases. Among the selected studies, they reported substantial heterogeneity and the absence of a standardized treatment protocol or methodology. This variability limited the ability to assess prognosis and hindered direct comparison between different techniques [9].

Their review concluded that, among 305 teeth treated with root amputation procedures, the failure rate—defined as tooth loss—was approximately 50%. However, only 42 of these teeth were treated exclusively for postendodontic indications [9]. Moreover, in the cases selected for postendodontic reasons, no detailed preoperative evaluation was reported that could distinguish between endodontic failure, vertical root fracture, or periodontal disease as the underlying cause [9].

Other review studies, such as those by Setzer et al. in 2012 [10] and Dommisch et al. in 2020 [11], encountered similar issues of heterogeneity in case classification and data comparison. In these studies, root amputation was generally performed to manage teeth with furcation involvement or severe periodontal compromise, rather than specifically for teeth with chronic apical periodontitis associated with an endodontic or postendodontic etiology due to vertical root fracture [10] [11].

In 2024, Ajlan [12] reported that the decision to perform root amputation was supported by long‐term follow‐up studies (Table 1) demonstrating favorable survival rates and predictable clinical outcomes over extended observation periods [12].

**Table 1:** Table adapted and modified from the review of previously published studies reported by Ajlan [12].

Author	Follow‐up period range in years (average)	No. of teeth	Overall percentage of teeth lost/failed
Bergenholtz, 1972 [13]	2–11	45	6
Klavan, 1975 [14]	1–7 (3)	34	2.9
Hamp and Nyman, 1974 [15]	5	87	0
Langer et al., 1981 [16]	10	100	38
Erpenstein, 1983 [17]	1–7 (2.9)	34	8.8
Buhler, 1988 [18]	10	28	32
Newell, 1991 [19]	0.5–7	70	30
Basten, 1996 [20]	2–23 (11.5)	49	8
Blomlof et al., 1997 [21]	3–10 (5.8)	146	22
Carnevale, 1998 [22]	10	175	7
Hou, 1999 [23]	5–13 (6.7 ± 1.9)	85	0
Park et al., 2009 [24]	10	342	29.8
Lee et al., 2012 [25]	10	149	59.7
Derks, 2018 [26]	4–30 (14.7 ± 6.8)	90	33.3
Alassadi, 2020 [27]	1–16.8 (5 ± 4.3)	85	44.7%
AlSayed, 2020 [28]	1–15 (9.62 ± 3.08)	130	43.1%

Therefore, root amputation can be considered a predictable treatment option with favorable long‐term outcomes. As illustrated in Figure 8, several teeth treated by conservative surgical approaches, including root amputation (1.6,2.6) and root hemisection (4.7), remained functional and in situ after more than 10 years of follow‐up.

**Figure 8:**
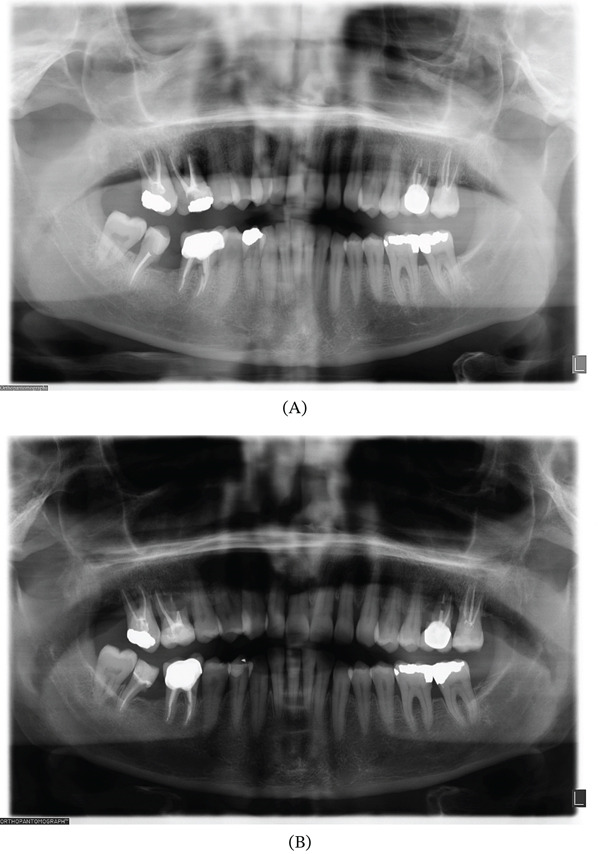
Figure illustrates a 14‐year follow‐up of a patient in whom all previously performed conservative surgical treatments remained functional and clinically stable, demonstrating the long‐term sustainability of these therapeutic interventions. (A) Panoramic radiograph taken on September 27, 2011, following a root hemisection of Tooth 4.7. (B) Panoramic radiograph taken on November 14, 2024, showing several remaining root resection treatments, including the 4.7 hemisection performed in 2011, mesiobuccal root amputation of Tooth 1.6 in 2014, and mesiobuccal root amputation of Tooth 2.6 in 2022.

Root amputation may provide a predictable long‐term outcome when the underlying pathology is accurately identified, and appropriate case selection criteria are fulfilled. Two factors appear to be essential for treatment success [10, 11]. First, the tooth should have received adequate endodontic therapy; when the quality of the previous root canal treatment is questionable, endodontic retreatment, either through a nonsurgical orthograde approach or surgical endodontic intervention, should be considered [10, 26, 28]. Second, long‐term success is highly dependent on achieving and maintaining periodontal stability through effective plaque control and regular supportive periodontal care.

The interpretation of survival and success rates reported for root amputation procedures remains challenging because this treatment modality is indicated for a wide spectrum of clinical conditions.

These include endodontic lesions associated with persistent apical periodontitis, periodontal defects affecting furcation‐involved teeth, combined endodontic–periodontal lesions, and, in selected cases, root fractures confined to a single root of a multirooted tooth [12, 26, 27]. As a result, the outcomes reported in the literature are heterogeneous and largely dependent on the underlying indication for treatment [10–12, 27].

Therefore, studies evaluating root resection should be interpreted according to the specific pathology being treated, as pooling different clinical scenarios may lead to inaccurate conclusions regarding prognosis and long‐term survival [8–12, 27, 28].

An additional indication that has received limited attention in the literature is the use of root amputation as a means of preserving alveolar bone prior to planned implant rehabilitation. Rather than proceeding directly to extraction, retention of the unaffected portion of the tooth may help maintain function and support the natural healing processes of the alveolar bone. This strategy could allow partial or complete regeneration of extraction‐related defects to facilitate future implant placement (Figure 9). Nevertheless, the available evidence remains scarce, and further clinical studies are required to determine the predictability and long‐term benefits of this therapeutic approach.

**Figure 9:**
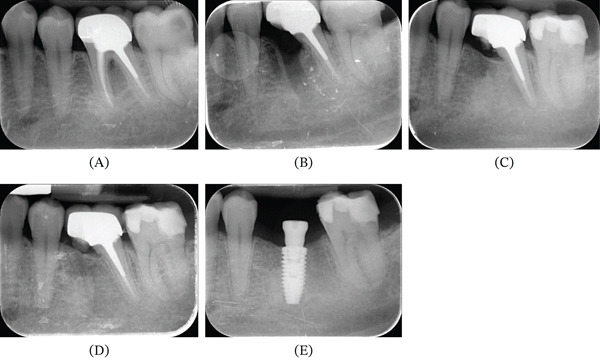
Radiographic follow‐up series of Tooth 3.6. (A) Radiograph taken in January 2008 showing a vertical root fracture of the mesial root. (B) November 2009: Root amputation of both the mesiobuccal and mesiolingual roots was performed. (C) February 2018: follow‐up radiograph. (D) July 2024: Fracture of the distal root occurs and extraction with implant placement is planned. (E) November 2024: Implant is successfully placed 15 years after the root amputation, without the use of adjunctive regenerative techniques.

The present case demonstrated a favorable clinical and radiographic outcome. The CBCT scan obtained at the 4‐year follow‐up showed evident bone regeneration, with improvements in both vertical height and horizontal width. It is well established that such procedures often present a questionable prognosis and may exhibit deterioration over time. Therefore, the availability of long‐term follow‐up data in this case provides valuable insight into the stability and predictability of the treatment. The positive outcome can be largely attributed to an accurate diagnosis based on clinical experience, despite the limited diagnostic resources available at the time of treatment, particularly the lack of CBCT imaging.

Within these limitations, the available evidence suggests that root resection remains a valuable tooth‐preserving procedure in carefully selected cases, allowing retention of strategically important teeth while maintaining function and supporting the preservation of the surrounding alveolar bone.

## 5. Conclusion

Root amputation is a complex procedure for which clear scientific evidence supporting its indication is limited, although it is also not contraindicated. It remains a valuable tooth‐preserving treatment option in carefully selected cases despite the limited availability of high‐quality evidence regarding its long‐term indications and outcomes. Accurate diagnosis and appropriate case selection are critical determinants of treatment success.

Furthermore, root resection may serve as a strategic and minimally invasive approach for preserving alveolar bone and maintaining function, potentially facilitating future implant rehabilitation while reducing the need for additional regenerative procedures.

## Funding

No funding was received for this manuscript.

## Conflicts of Interest

None of the authors have a conflict of interest to disclose.

## Data Availability

The data that support the findings of this study are available from the corresponding author upon reasonable request.
